# High drug resistance levels could compromise the control of HIV infection in paediatric and adolescent population in Kinshasa, the Democratic Republic of Congo

**DOI:** 10.1371/journal.pone.0248835

**Published:** 2021-04-15

**Authors:** Marina Rubio-Garrido, Gabriel Reina, Adolphe Ndarabu, Ana Rodriguez-Galet, Ana Valadés-Alcaraz, David Barquín, Silvia Carlos, África Holguín

**Affiliations:** 1 HIV-1 Molecular Epidemiology Laboratory, Microbiology and Parasitology Department, Hospital Ramón y Cajal-IRYCIS and CIBEREsp-RITIP-CoRISPe, Madrid, Spain; 2 Microbiology Department, Clínica Universidad de Navarra, Navarra Institute for Health Research, Institute of Tropical Health, Universidad de Navarra, Pamplona, Spain; 3 Monkole Hospital, Kinshasa, Democratic Republic of Congo; 4 Department of Preventive Medicine and Public Health, Universidad de Navarra, Navarra Institute for Health Research, Institute of Tropical Health, Universidad de Navarra, Pamplona, Spain; University of Cincinnati College of Medicine, UNITED STATES

## Abstract

**Background:**

The inadequacy of HIV viraemia and resistance monitoring in Africa leads to uncontrolled circulation of HIV strains with drug resistance mutations (DRM), compromising antiretroviral therapy (ART) effectiveness. This study describes the DRM prevalence and its therapeutic impact in HIV-infected pediatric patients from Kinshasa (Democratic Republic of Congo, DRC).

**Methods:**

From 2016–2018, dried blood were collected from 71 HIV-infected children and adolescents under ART in two hospitals in Kinshasa for HIV-1 DRM *pol* analysis, predicted ARV-susceptibility by Stanford and phylogenetic characterization.

**Results:**

HIV-1 sequences were recovered from 55 children/adolescents with 14 years of median-age. All had received nucleoside and non-nucleoside reverse transcriptase inhibitors (NRTI, NNRTI), 9.1% protease inhibitors (PI) and only one integrase inhibitor (INI). Despite the use of ART, 89.1% showed virological failure and 67.3% carried viruses with major-DRM to one (12.7%), two (47.3%), or three (5.5%) ARV-families. Most children/adolescents harbored DRM to NNRTI (73.5%) or NRTI (61.2%). Major-DRM to PI was present in 8.3% and minor-DRM to INI in 15%. Dual-class-NRTI+NNRTI resistance appeared in 53.1% of patients. Viruses presented high/intermediate resistance to nevirapine (72.9% patients), efavirenz (70.9%), emtricitabine/lamivudine (47.9%), rilpivirine (41.7%), etravirine (39.6%), doravidine (33.3%), zidovudine (22.9%), among others. Most participants were susceptible to INI and PI. Great diversity of variants was found, with a high rate (40%) of unique recombinants.

**Conclusion:**

The high DRM prevalence observed among HIV-infected children and adolescents in Kinshasa could compromise the 95-95-95-UNAIDS targets in the DRC. It also reinforces the need for routine resistance monitoring for optimal rescue therapy election in this vulnerable population to control the spread of resistant HIV in the country.

## Introduction

Over the last years, scale-up of HIV antiretroviral treatment (ART) has had a major impact on HIV-related illness, averting AIDS-related deaths, preventing new HIV infections, and resulting in cost savings [1]. Despite significant advances in the prevention and treatment of HIV, countries continue to experience serious gaps in ART service delivery, including suboptimal treatment and care services, drug stock-outs, limited or absent access to routine viral load quantification for ART monitoring, and inadequate adherence to ART, which benefit the emergence and transmission of HIV drug resistance (HIVDR) [2]. As efforts to scale-up treatment continue, and more individuals receive antiretroviral drugs (ARV) for HIV treatment or prevention, a further increase in levels of HIVDR will likely compromise the substantial gains already achieved in the HIV response [3], and the 95-95-95 UNAIDS targets for 2030 [4] in many countries.

Since non-nucleoside reverse transcriptase inhibitors (NNRTI) based ART continues to be used in first-line ART in the DRC as in many Sub-Saharan Africa countries, an increase in pre-antiretroviral-treatment drug resistance (PDR) to NNRTI over 10% is expected in this region over the next 15 years. This PDR rate will be responsible for 16% of AIDS deaths and 9% of new HIV infections in Sub-Saharan Africa in 2016–2030 [5]. Individuals with PDR to NNRTI who initiate an NNRTI-based regimen are less likely to achieve viral load suppression, more likely to have virological failure, and to discontinue treatment. This pattern is also observed in treated HIV-infected children [6]. For women living with HIV initiating ART during pregnancy, resistance poses a significant challenge for the elimination of mother-to-child transmission of HIV [7]. DRM to reverse transcriptase inhibitors are more common in low-middle income countries, where these ARV are provided as a first-line regimen, regardless of the presence of HIVDR or prior exposure to ARV. Besides, the significant loss in susceptibility of the nucleoside reverse transcriptase inhibitors (NRTI) class is of particular concern for young children, for whom the number of licensed NRTIs is limited [3]. Therefore, the emergence of HIVDR threatens the improvements in morbidity and mortality anticipated by a “treat all” approach and even the scale-up of pre-exposure prophylaxis [8].

Nearly 1.7 million children, 1.6 million adolescents, and 3.5 million young people are currently infected with HIV-1 worldwide, 90% living in Sub-Saharan Africa [9]. Adolescents are highly vulnerable to HIV infection, mainly those living in settings with a generalized HIV epidemic [10]. The WHO estimates that one-seventh of all new HIV infections occur during adolescence [11]. ART has strikingly reduced morbidity and mortality in children and adolescents. However, suboptimal virological suppression fosters the emergence of viruses carrying HIV drug resistance mutations (DRM), with important consequences for children and adolescents as they require ART for longer periods than adults [12]. HIV-infected children are at high risk of acquiring drug-resistant viruses, which is of particular concern in settings where ARV drug options are limited. High levels of acquired drug resistance after the first treatment reduce the virus’ susceptibility to ARVs and jeopardize the recycling of ARVs in second-line ART [3].

In the Democratic Republic of Congo (DRC), the coverage of people living with HIV receiving ART was 57% in 2018, but only 25% among children [9], although more than 100,000 children and adolescents are HIV-infected in the DRC. Furthermore, viraemia and HIVDR monitoring for ART optimization are absent during the clinical routine in all HIV-infected subjects. It can lead to late treatment failures identification due to resistant viruses, to empirical switches of ART regimens with recycled NRTIs, and to resistant viruses spreading. Without correct HIV monitoring, patients may spend months or even years on a failing ARV regimen, resulting in DRM accumulation and increased rates of morbidity and mortality [13–16]. Accordingly, the Joint United Nations Program on HIV/AIDS (UNAIDS) and the WHO reinforced the importance of HIVDR monitoring to control the HIV epidemic [3, 4, 17], mainly in key populations such as infants and adolescents.

For all these reasons, the aim of this study is to analyze HIVDR prevalence and its therapeutical impact on the children and adolescent population from Kinshasa (DRC). To find out the current situation of these populations in the DRC is particularly important due to their lifelong-treatment with more years under ART than adults.

## Methods

### Samples collection

Dried blood specimens (DBS) from HIV-infected children and adolescents under clinical follow-up in paediatric units at Monkole and Kalembelembe Hospitals in Kinshasa (DRC) were collected in 2016–2018. DBS samples were prepared by spotting 70 μl of venous blood collected by venipuncture in EDTA-anticoagulant tubes into each dot on a Whatman 903 Protein Saver Card (Schleicher&Schuell, Dassel, Germany). They were dried separately overnight at room temperature, sealed in a zip-lock bag with desiccant, and stored at −20°C until transported in dry ice to the HIV-1 Molecular Epidemiology Laboratory in Madrid, Spain, where samples were stored at −80°C until further analysis.

### Resistance analysis

RNA was extracted from two DBS dots using the NucliSENS EasyMag automated platform (BioMerieux) following elution with lysis buffer (EasyMag). Viral RNA was amplified in the HIV-1 *pol* coding region by RT-PCR and nested-PCR using primers designed by WHO [18] for protease (PR) and reverse transcriptase (RT) regions, as previously described [19]. We used ANRS primers [20] for viral integrase (IN) amplification. Viral sequences included the complete HIV-1 PR (codons 1–99), partial RT (1–335), and IN (1–285). PCR amplicons were purified using the Illustra™ ExoProStar 1-Step™ (GE Healthcare Life Sciences, Little Chalfont, UK) and sequenced by Macrogen Inc. (Gasan-dong, Geumchun-gu, Seoul, Korea). Sequences were assembled and manually edited using Lasergene software. DRM in pretreated children/adolescents to PR inhibitors (PI), NRTI, NNRTI, and integrase inhibitors (INI) were characterized by Stanford HIVdb Program v8.8 (Stanford University, Palo Alto, CA, USA) as well as predicted resistance level to each ARV.

### Statistics analysis

Descriptive statistical analysis was performed calculating mean, median, and interquartile range (IQR). The percentage of virus carrying DRM was calculated with 95% confidence intervals (CI). The statistical significance was calculated using a T-Student or Mann–Whitney U tests. P-values <0.05 were considered statistically significant. Statistical analyses were conducted using GraphPad Prism version 8.0.1 (San Diego, CA, USA).

### HIV-1 variant characterization

Nucleotide sequences were translated and aligned using the ClustalW algorithm implemented in MEGA6. For HIV-1 variant characterization we used reference sequences available in Los Alamos HIV Sequence Database (http://www.hiv.lanl.gov). Phylogenetic trees (PhyML tree) were reconstructed by maximum-likelihood (ML) method [21] using the general time reversible plus proportion of invariable sites plus gamma distribution parameter (GTR+I+G) evolutionary model. For estimating the bootstrap values on the inferred PhyML tree topology, Shimodaira-Hasegawa test using FastTree program was used (support >90%) [22]. Sequences not clustering with any known subtype or circulating recombinant form (CRF) were analyzed using Recombination Detection Program (RDP3v4.13) [23], identifying the subtypes involved in eventual recombination events and hypothetical recombination breakpoints. To further confirm the detected putative recombination events, new phylogenetic analyses were performed using the sequence fragments assigned to different subtypes according to the proposed breakpoint position(s) defined by RPD3. In the positive cases, the recombinant sequences were redefined as unique recombinant forms (URFs). The remaining cases were denoted as unique unclassified (U) variants.

### Accession numbers

HIV-1 sequences were submitted to GenBank with the following accession numbers: MH920378-MH920389, MN530990-MN530996, MN531052-MN531082 and MN998519-MN998523.

### Ethical aspects

The project was approved by the Human Subjects Review Committees at Monkole Hospital/University of Kinshasa (DRC) and University Hospital Ramón y Cajal (Madrid, Spain). Informed consent was obtained from parents or guardians of enrolled participants. Children and adolescents also provided assent after parental consent when they could understand the meaning of participation in the study. All methods were carried out according to relevant guidelines and regulations.

## Results

### Study population

DBS samples were collected between 2016 and 2018 from 71 HIV-positive subjects (34 children, 37 adolescents) under HIV care and ART in paediatric units of Monkole and Kalembelembe Hospitals. Among them, 85.5% of children/adolescents presented viraemia higher than 1,000cp/ml at sampling. HIV-1 sequences could be recovered from 55 (77.5%) patients in, at least, one genotype region for DRM analysis. (**Table 1**).

**Table 1:** Demographic and virological characteristics of children and adolescents of the study cohort with available HIV sequence.

	Children [0–14] (%)	Adolescents [15–21] (%)	P value	Total cohort (%)
**Total available sequences**	27 (100)	28 (100)		55 (100)
**Female**	12 (44.4)	17 (60.7)		29 (52.7)
**Median age (years)**				
**At HIV diagnosis in the DRC [IQR]**	4 [1–8]	10.5 [5.3–13]	**	6 [2–12]
**At first ART experience[IQR]**	4 [0.8–8]	12 [7–13]	**	7 [3.5–12]
**At DBS collection [IQR]**	11 [9–12]	16 [15–17]	**	14 [11–16]
**HIV status in mothers**				
**HIV+**	12 (44.4)	7 (25)		19 (34.6)
**HIV-**	2 (7.4)	5 (17.9)		7 (12.7)
**unknown**	13 (48.2)	16 (57.1)		29 (52.7)
**HIV-1 viraemia**				
**>1,000c/ml by Roche VL**	25 (92.6)	24 (85.7)		49 (89.1)
**ART exposure at sampling**				
**ART**	27 (100)	28 (100)		55 (100)
**Median time under ART [IQR]**	6 [1–8]	6 [1.3–10]		6 [1–8.5]
**Mean ARV exposure time (years)**				
**To NRTI**	5.2 (0–12.2)	6.2 (0–13.3)		5.8 (0–13.3)
**To NNRTI**	4.6 (0–11.2)	6.2 (0–13.3)		5.4 (0–13.3)
**To PI**	0.9 (0–12.2)	0.26 (0–3.4)		0.6 (0–12.2)
**To INI**	0	0.09 (0–2.7)		0.05 (0–2.7)
**Number of different ART regimens until sampling**				
**1**	13 (48.1)	6 (21.4)	*	19 (34.5)
**2**	7 (25.9)	10 (35.7)		17 (30.9)
**3**	5 (18.5)	8 (28.6)		13 (23.6)
**4**	1 (3.7)	1 (3.6)		2 (3.6)
**5**	1 (3.7)	2 (7.1)		3 (5.5)
**7**	0	1 (3.6)		1 (1.8)
**NRTI experience**				
**3TC**	27 (100)	28 (100)		55 (100)
**AZT**	21 (77.8)	26 (92.9)		47 (85.5)
**TDF**	13 (48.1)	23 (82.1)	*	36 (65.5)
**DDI**	1 (3.7)	0		1 (1.8)
**ABC**	5 (18.5)	2 (7.1)		7 (12.7)
**D4T**	0	4 (14.3)	*	4 (7.3)
**NNRTI experience**				
**NVP**	21 (77.8)	25 (89.3)		46 (83.6)
**EFV**	13 (48.1)	23 (82.1)	*	36 (65.5)
**PI experience**				
**LPV/r**	3 (11.1)	2 (7.1)		5 (9.1)
**INI experience**				
**DTG**	0	1 (3.4)		1 (1.8)
**HIV+ subjects with available *pol* HIV-1 sequences**				
**PR**	18 (66.7)	18 (64.3)		36 (65.5)
**RT**	23 (85.2)	26 (92.9)		49 (89.1)
**INT**	22 (81.5)	18 (64.3)		40 (72.7)
**HIV-1 variants**				
**Non-B pure subtypes**	15 (55.6)	14 (50)		29 (52.8)
**CRF**	1 (3.7)	1 (3.6)		2 (3.6)
**URF**	9 (33.3)	13 (46.4)		22 (40)
**U**	2 (7.4)	0		2 (3.6)

Data according to clinical reports. DRC, the Democratic Republic of Congo; DBS, dried blood Spot; ART, antiretroviral treatment; NRTI, nucleoside transcriptase reverse inhibitor; NNRTI, non-NRTI; PI, Protease inhibitor; INI, integrase inhibitors; IQR; *interquartile range*; c/ml, copies of HIV-1 RNA per milliliter; VL, viral load; ART, antiretroviral therapy; ARV, antiretroviral drug; 3TC, lamivudine; AZT, Zidovudine; TDF, Tenofovir; DDI, Didanosine; ABC, Abacavir; D4T, Stavudine; NVP, Nevirapine; EFV, Efavirez; LVP/r, Lopinavir/Ritonavir; DTG, Dolutegravir; PR, protease; RT, reverse transcriptase; IN, integrase; CRF, circular recombinants forms; URF, unique recombinants forms; U, unknown.

**, *p<0*.*001;*

*, *p<0*.*05*. Viral load quantified by Roche Cobas v2.0, Limit of quantification <20cp/ml. Corrected cp/ml plasma considering hematocrit [24]

**Table 1** describes the main features of the study population. The median age at diagnosis/first ART of the 55 subjects with a sequence was 6 [IQR:2–12]/7 [IQR:3.5–12] years. The median age at study enrolment was 14 [IQR: 11–16] years. All of them were under ART at sampling and had received NRTI and NNRTI, 9.1% were PI-experienced, and only one adolescent (1.8%) had received INI.

The 55 participants with available viral sequence were under 1^st^ (34.5%), 2^nd^ (30.9%) or 3^rd^ ART regimen (23.6%) at sampling, and 6 (10.9%) had received 4, 5 or 7 different ARV regimens. The most common regimen was zidovudine/lamivudine with nevirapine (81.8%) or efavirenz (16.4%), followed by tenofovir/lamivudine/efavirenz (63.6%). All subjects were lamivudine-experienced, followed by zidovudine (85.5%), nevirapine (83.6%), tenofovir (65.5%), and efavirenz (65.5%).

Although children and adolescents had both been under ART exposure for 6 years [IQR: 1–8.5], the number of children under 1^st^ ARV regimen was significantly higher than in adolescents (p<0.05). Moreover, 89.1% of the 55 children/adolescents under study presented virological failure at sampling. The 55 samples showed median viral load of 11,824cp/ml [IQR: 2,950–59,578].

Moreover, adolescents presented a significantly higher delay in HIV diagnosis than children (10.5 *vs*. 4 years) and 1^st^ ART (12 *vs*. 4 years). Besides, adolescents showed higher exposure to several drugs than children, including tenofovir, stavudine and efavirenz (**Table 1**). Regarding HIV-1 variants, 52.8% children/adolescents carried pure subtypes at *pol* (13A, 1A2, 1B, 3C, 1D, 1F1, 6G, 2H, 1J) and 43.6% recombinants, being mainly URF (40%). CRF included 1 CRF27_cpx and 1 CRF47_BF. The remaining variants were unclassified (U) variants (**Table 1**).

### HIVDR among children and adolescents on ART

**Fig 1:**
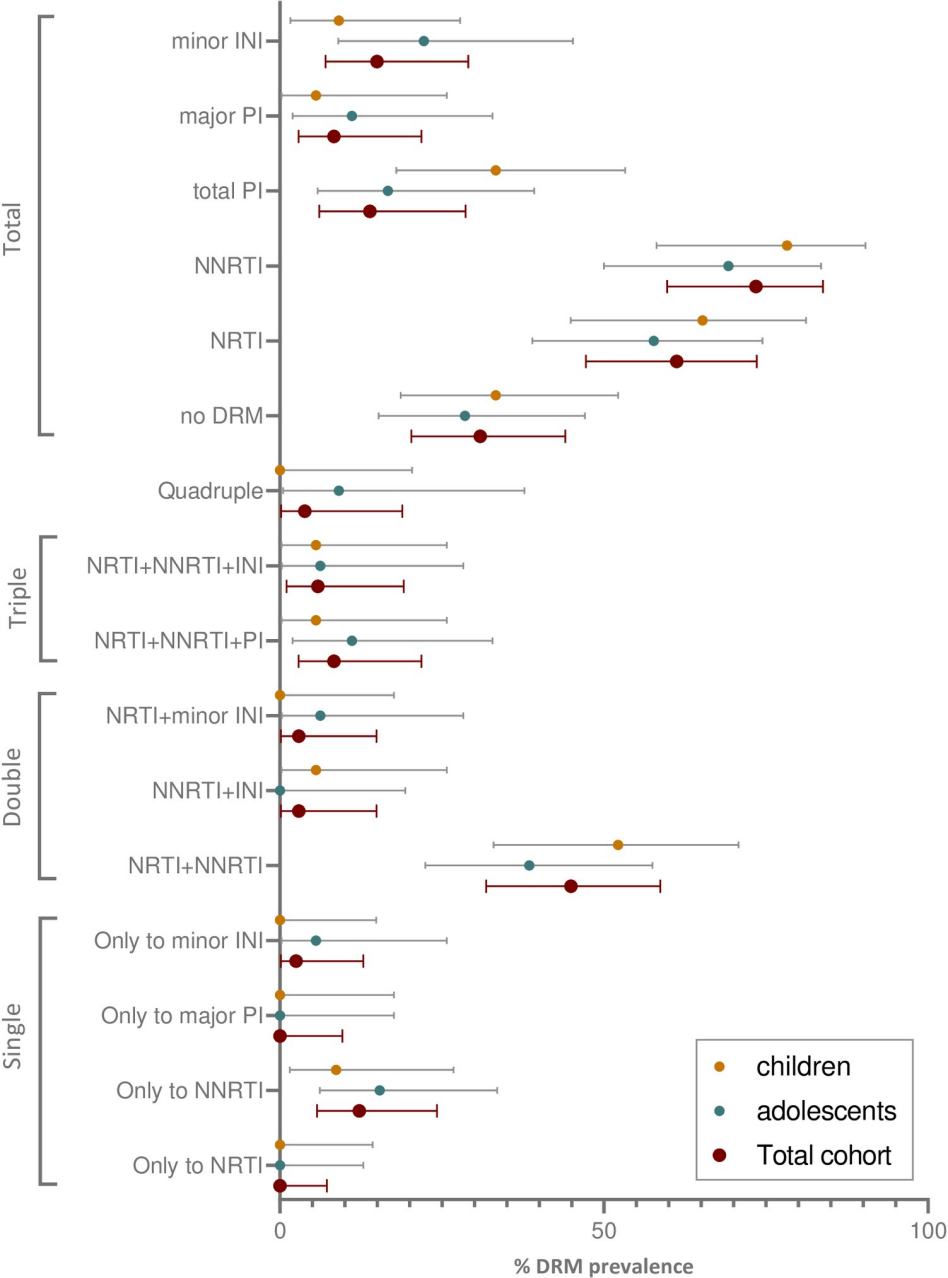
Rate of patients carrying DRM to the main ARV families at study population. Mean DRM prevalence (colored dots) and 95% confidence intervals on the study cohort with available sequence by age. Single resistance, to one ARV family; double, triple, or quadruple resistance and total DRM ARV families. DRM to PI are always major unless otherwise indicated. DRM to INI are always minor. ARV, antiretroviral drugs; NRTI, nucleoside transcriptase reverse inhibitor; NNRTI, non-nucleoside transcriptase reverse inhibitor; PI, Protease inhibitor; INI, integrase inhibitors. Rates calculated considering available sequences (55) reported in **Table 1**. More data available in **S1 Table**.

**Fig 1** shows the drug resistance mutations to the main ARV families found in the study cohort. Among the 55 children and adolescents with available *pol* sequences, 37 (67.3%) were infected with viruses carrying major-DRM to one (12.7%), two (47.3%) or three (5.5%) ARV families, without significant differences among children and adolescents’ groups. Only a third (32.7%) of the treated study cohort did not present major-DRM at sampling, despite having virological failure with no suppressed viral load in 88.2% of them. When also considering minor-DRM to PI and INI, 38 (69.1%) were infected with viruses carrying DRM to one (12.7%), two (43.6%), three (10.9%), or four (1.8%) ARV families (**Fig 1** and **S1 Table**).

Of the 36/49/40 successfully sequenced samples at PR/RT/IN, DRM to NNRTI were present in 73.5% [95% CI, 59.7–83.8], DRM to NRTI in 61.2% [95% CI,47.2–73.6], major-DRM to PI in 8.3% [95% CI,2.8–21.8] and minor-DRM to INI in 15% [95% CI, 7.1%-29.1]. Dual-class NRTI+NNRTI resistance was present in 44.9% [95% CI, 31.9–58.7] children/adolescents (**S1 Table**). All viruses harboring DRM to NRTI also contained DRM to NNRTI. Only one 15.5 years old adolescent with 13.3 years of ART experience carried viruses with resistance to the 4 drug families, although with minor DRM to INI.

**Fig 2:**
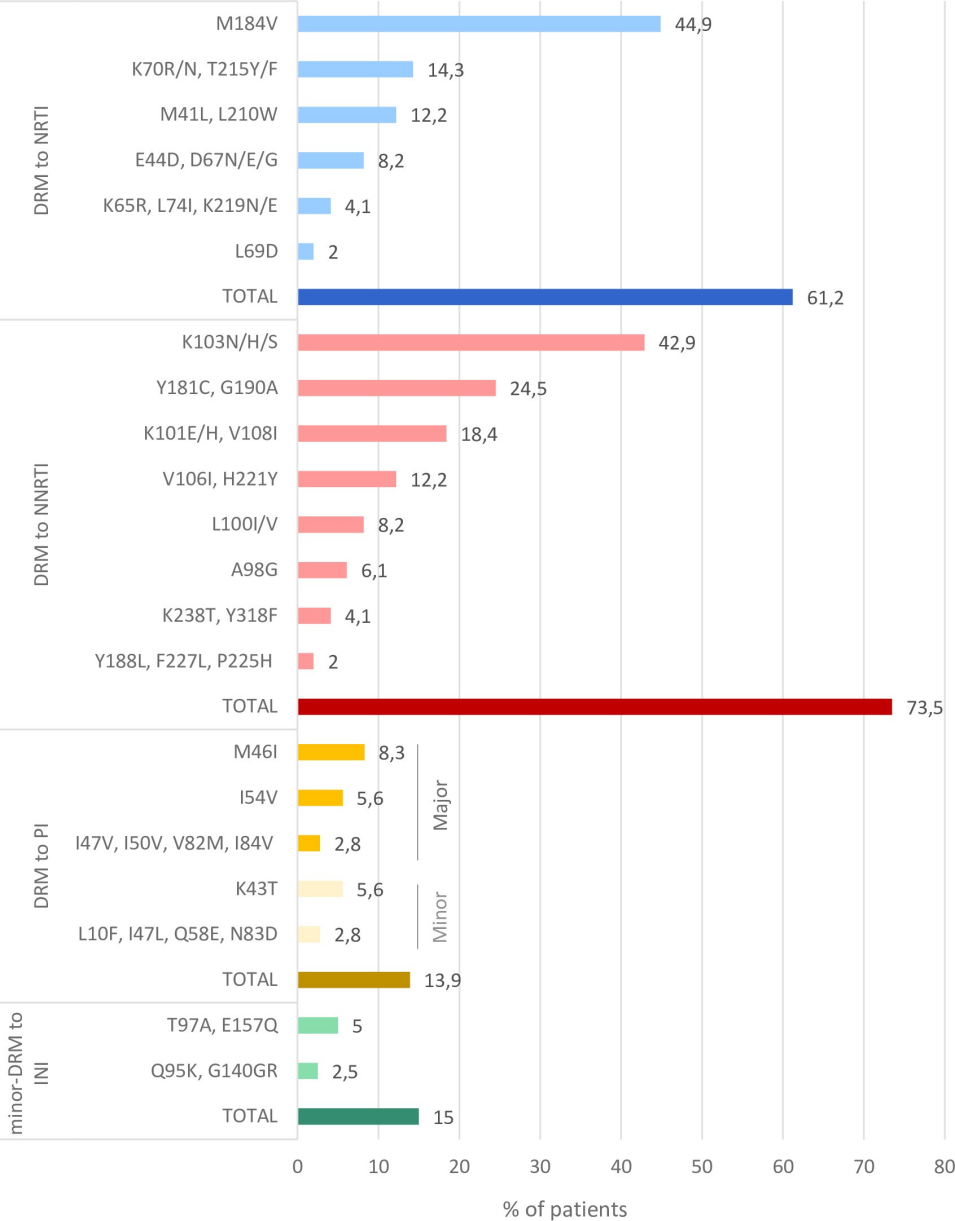
Drug resistance mutations to the main antiretroviral families in the study population. DRM, drug resistance mutation; NRTI, nucleoside transcriptase reverse inhibitor; NNRTI, non-NRTI; PI, Protease inhibitor; INI, integrase inhibitor. Available sequences in 55 children/adolescents under study: 38PR, 49RT, and 40IN. More data available in **S2 Table**.

**Fig 2** and **S2 Table** show the DRM found in the study population. DRM present in 61.2% of children/adolescents were to NRTI, M184V (44.9%), K70R/N and T215Y/F (14.3% each), M41L/L210W (12.2% each). DRM to NNRTI were presented in 73.5% of population: K103N/H/S (42.9%), Y181C/G190A (24.5% each), V108I/K101E/H (18.4% each) and H221Y/V106I (12.2% each). The three subjects with major DRM to PI carried M46I (8.3%) and two also had I54V, only one of them had previous experience to PI. Minor DRM to INI were found in 15% of subjects with available IN sequences, 9.1% children, and 22.2% adolescents. When comparing the rate of infections with resistant viruses carrying major DRM in children (0–14 years) *vs*. adolescents (15–21 years) under study, no significant difference was observed (**S1 Table**).

**Fig 3:**
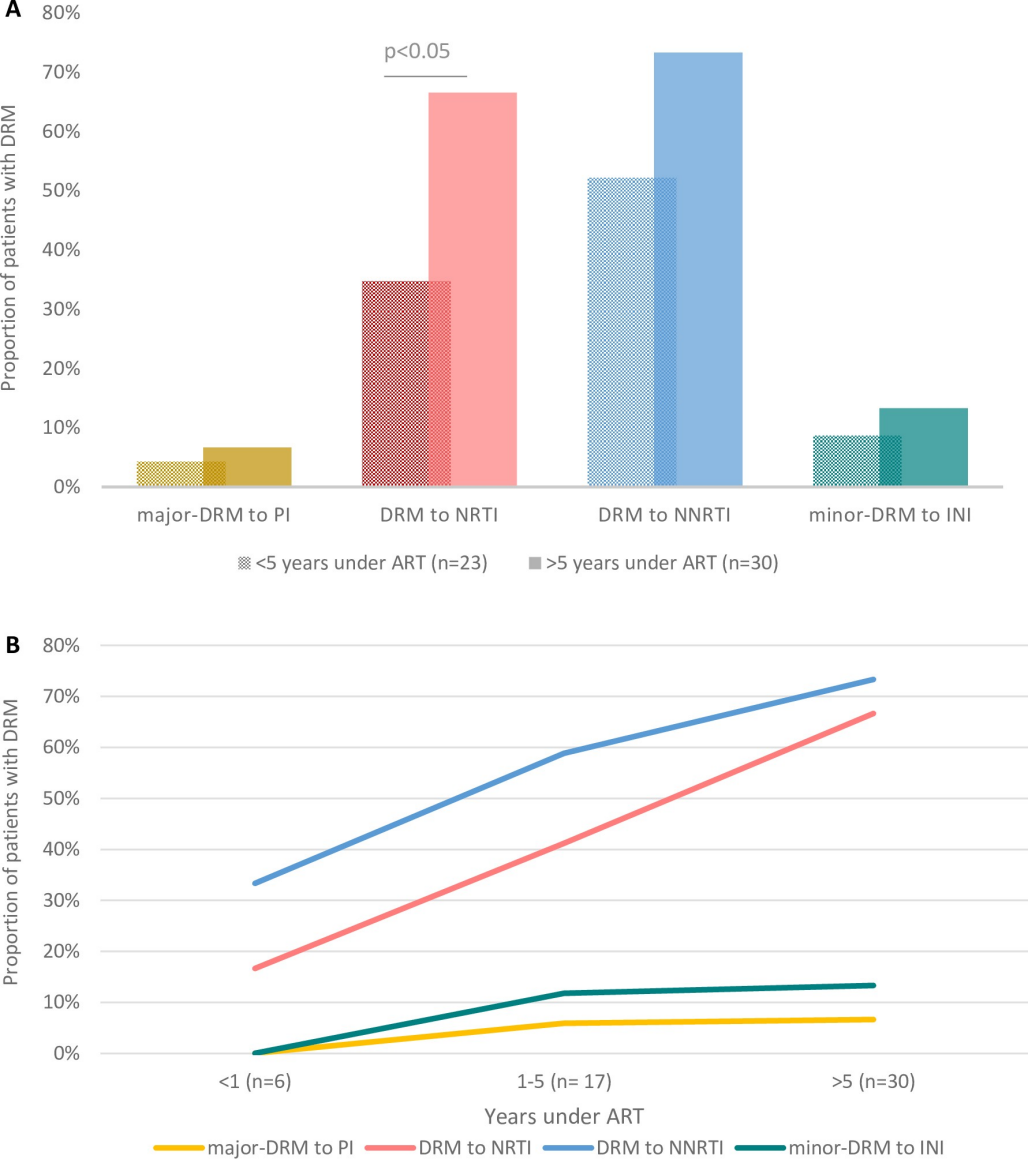
HIV-infected patients carrying DRM (A) and trends (B) according to ART exposure time in the study population. ART, antiretroviral therapy; DRM; drug resistance mutation; NRTI, nucleoside reverse transcriptase inhibitor; NNRTI, non-nucleoside reverse transcriptase inhibitor; PI, protease inhibitor; INI, integrase inhibitor; n, number of patient. In 2 of the 55 subjects time under ART was unknown.

Infections with resistant viruses were associated with ART exposure time. DRMs to all ARV families were higher in those patients having been under ART for more than 5 years (**Fig 3A**). The rate of children and adolescents carrying viruses resistant to NRTI was 2 times higher in those having been under ART for more than 5 years (OR = 3.75; 95% CI [1.22–11.34], p<0.05) (**Fig 3A**). **Fig 3B** shows the steadily tend to increase of infections with resistant viruses with ART exposure time.

### Predicted ARV susceptibility

**Fig 4:**
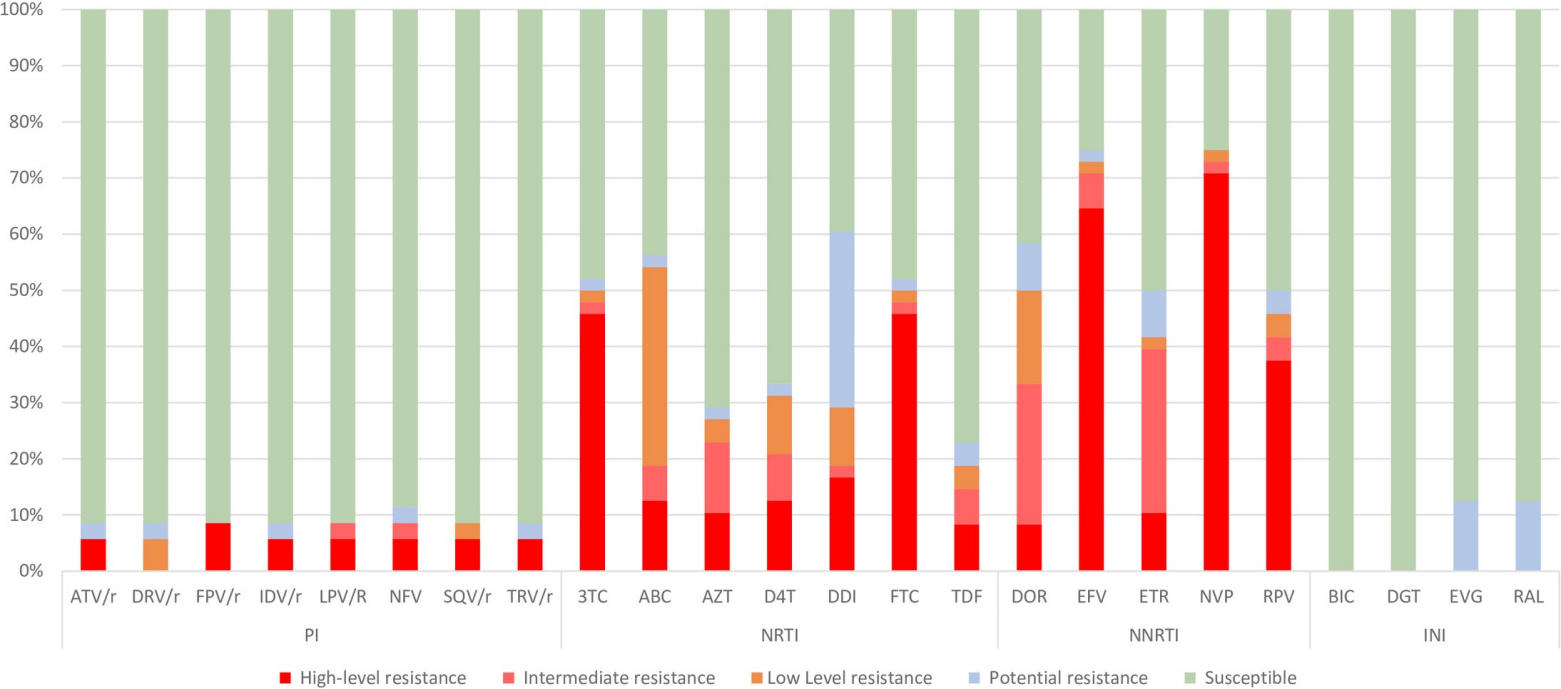
Predicted ARV susceptibility by Stanford in patients with available sequence. Predicted ARV susceptibility in 38PR/49RT/40IN available sequences from 55 children/adolescents under study. NRTI, nucleoside reverse transcriptase inhibitor; NNRTI, non-nucleoside reverse transcriptase inhibitor; PI, protease inhibitor; INI, integrase inhibitor; ATV/r, Atazanavir/Ritonavir; DRV, Darunavir; FPV, Fosamprenavir; IDV/r, Indinavir/Ritonavir; LVP/r, Lopinavir/Ritonavir; NFV, Nelfinavir; SQW, Saquinavir; TPV/r Tipranavir/Ritonavir; ABC, Abacavir; AZT, Zidobudine; D4T, Estavudine; DDI, Didanosine; FCT, Emtricitabine; 3TC, Lamivudine; TDF, Tenofovir; DOR: Doravirine; EFV, Enfavirez; ERT, Etravirine; NVP, Nevirapine; RPV, Rilpivirine; DGT, Dolutegravir; EVG, Elvitegravir; RAL, Raltegravir. Information per patient in **S1 Fig**.

Most children and adolescents with available *pol* genotype were infected with viruses susceptible to PI (88.9%) and INI (87.5%). A high resistance level was observed in 70.8% subjects to nevirapine, in 64.6% to efavirenz, in 45.8% to emtricitabine, or lamivudine, 37.5% to rilpivirine, and at lower rates for other ARV, except for INI (**Fig 4** and **S1 Fig**).

When considering both high or intermediate resistance level to NNRTIs, most (72.9% and 70.9%) subjects presented high and intermediate resistance to nevirapine and efavirenz, respectively, and 41.7% to rilpivirine, 39.6% to etravirine, and 33.3% to doravidine. For NRTI, half (47.9%) of the subjects carried viruses with high and intermediate resistance to lamivudine and emtricitabine, 22.9% to zidovudine, 18.8% to abacavir, and 14.6% to tenofovir. For PI, these rates were lower than 10% and absent for INI **(Fig 4 and S1 Fig)**.

## Discussion

This study represents the first characterization of HIV drug resistance among HIV-infected children and adolescents in the DRC. Moreover, it provides the most recent data of resistance to ARV drugs in Kinshasa and the DRC. While all published studies from DRC reported resistance data in naïve [25–28] or treated adults [25, 29] with samples collected from 2002 to 2014, our study had analyzed samples collected from 2016–2018. In addition, the present study is the first reporting resistance data for INI in that country, which is appropriate before the expected broad implementation of dolutegravir implementation in the DRC.

In the DRC and other Sub-Saharan Africa countries, HIV monitoring is limited due to a weak Public Health System and administration. Molecular diagnosis and VL testing are poorly accessible, mainly centralized in a national laboratory or restricted to some specific private hospitals. Moreover, resistance monitoring is not routinely available in the DRC [30]. For these reasons, ART regimen changes are determined by ARV availability, sometimes restricted by stock-outs [31], toxicity, and secondary effects in patients. No antiretroviral stock out was reported in Monkole and Kalembelembe hospitals during the study period.

According to the published data in samples from ART-naïve adults collected from 2002–2014 in DRC, the reported pre-antiretroviral-treatment drug resistance (PDR), a predictor of treatment failure, ranged from 0%-2.4% to PI, from 0–18.3% to NRTI, and from 0–9.8% to NNRTI, and unknown for INI [25–28]. Our study in children and adolescents from Kinshasa revealed that 7 out of 10 participants with available sequences harbored DRM, 4 out of 10 double resistance to NRTI+NNRTI, and 1 in 10 triple resistance to NRTI+NNRTI+PI. Of note, 7 out of 10 subjects with DRM to NNRTI had intermediate or high-level resistance to efavirenz and nevirapine, ARVs involved in first-line ART in the DRC. The observed burden of major DRM to PI in treated pediatric population was substantially higher than that previously reported in HIV-infected treated adults in the country [25, 26, 29]. Although adherence was promoted by medical staff, as well as in group of support with peers, accordingly to local staff, a third of 55 ART-treated children and adolescents with available sequence presented unsuppressed viraemia in the absence of resistant viruses. It would suggest that adherence support is required in this pediatric collective [3].

Since 2013, the WHO has recommended using PI-based ART regimens for children. In 2018, the WHO formally encouraged the phase-out of NNRTIs across age groups, with the introduction of dolutegravir for children with approved dose. However, in 2017, globally nearly 77% of children were still receiving nevirapine in first-line ART due to limited supplies of child-friendly drug formulations [3, 32]. Previous national guidelines from ART in the DRC recommended tenofovir + lamivudine + efavirenz as the first-line regimen for infected adolescents and adults, tenofovir + lamivudine + ritonavir-boosted lopinavir for second-line, and abacavir + dolutegravir + ritonavir-boosted darunavir for the third-line ART. In infants, guidelines recommended abacavir + lamivudine + ritonavir-boosted lopinavir or efavirenz. Dolutegravir, included in 2019 in the first-line ART therapy in children, adolescents and adults on the DRC will probably improve viral load suppression, and it is expected to reduce mortality and HIV incidence compared to EFV-based regimens [3]. However, it is important to monitor their implementation and to forecast the development of DRM [33]. INI treatment would therefore be a good alternative to NNRTIs due to the high presence of DRM to this ARV family, as we observed in our study. Moreover, the absence of major DRM to INI in the study population (never exposed to this drug family, except in one case), would strongly support the implementation of new dolutegravir-based treatment in the DRC. However, the circulation of viruses with minor DRM to INI residues (observed in 15% of children/adolescents) could affect INI susceptibility in combination with other substitutions [34]. If the presence of M184I/V or K65R (DRM to NRTI) could prevent dolutegravir resistance as previously suggested [35], it should be explored further. As expected, the great diversity of HIV-1 variants in the country [36] is reflected in our study cohort, with a high rate of URF.

A limitation of this study is that samples were collected for ART treated subjects in two hospitals in Kinshasa, and it could not be representative of the situation on a city or country-wide level. However, Monkole and Kalembelembe hospitals are considered in the top 5 of clinical centers with more HIV-infected children and adolescents under ART in Kinshasa. Further analyses are required to monitor the current transmission of drug-resistant strains in ART-naive HIV-infected children and adolescents in the DRC.

The sample size, although modest, was similar to other related resistance studies among treated patients in the DRC, analyzing from 55 [29] to 93 [25] *pol* sequences. Furthermore, the presented data are the first resistance information available for children and adolescents under ART in the country. Previous reports showed that HIVDR in naïve adults was very low in the DRC before the year 2007 [26, 27], although some TDR in some RT residues reached prevalence higher than 10% in samples collected in Kinshasa during 2013–2014 [28]. Understanding levels of DRM prior to treatment initiation is particularly important in children because they have higher viraemias and faster disease progression compared to adults [37]. However, it would be necessary to know the TDR rate in ART-naïve children and adolescents in the city and country. Unfortunately, we cannot estimate the rate of resistant viruses which could have been transmitted at first HIV infection in our study cohort. The third limitation was the lack of complete information regarding HIV-status, treatment, and resistance data from all their mothers, which could have identified cases of vertical DRM transmission. The last limitation would be the absence of resistance data in 16 ART-treated participants with negative *pol* amplifications. It could be explained by the low viraemias reported in some of them due to ART control. Moreover, we cannot exclude that some negative PCR could be due to the high viral genetic variability in viral targets for primers used for *pol* amplication. A recent study reported the temporal trends of HIV-1 subtypes and recombinants in the DRC during a 43-year period (1976–2018) and in Kinshasa from 1983 to 2018, showing a high number of different HIV-1 variants currently circulating in that city, with an increase of complex and unique recombinants in the last years [38].

Multiple studies have demonstrated that the percentage of patients with drug resistance goes up steadily as time on treatment increases [25, 39], as we have observed in the current study. Since children and adolescents are patients with a lifelong-treatment with more years under ART than adults, the study of DRM is particularly important in pediatric populations.

Thus, all generated results were communicated to clinicians in Kinshasa for better care of pediatric patients under study, providing useful data for ART regimen optimization. This work provides unique information related to these vulnerable populations in Kinshasa and in the rest of the country, where nearly 3 million children and adolescents are HIV infected without VL and resistance monitoring. We also reinforced the use of DBS as field-friendly and an useful specimen to carry out resistance analysis in low-middle income countries as the WHO recommends in the absence of plasma [3].

DRM monitoring is also crucial to control infection in these countries, where most paediatric HIV occur. Also, it is important to reinforce adherence support, and to implement routine VL quantification and resistance testing in the DRC national guidelines, strengthening the country’s laboratory services. Our findings also support alternative ART regimens based on PI and INI instead of RTI in HIV-infected children and adolescent population in that country. These improvements would help to control the spread of resistant viruses among new HIV infections, getting the DRC closer to WHO’s 95-95-95 targets [40].

## Supporting information

S1 Table Patients carrying DRM to ARV families at study population: Children (0–14 years), adolescents (15–21 years) and total. (PDF)

S2 Table Drug resistance mutations in children (0–14 years), adolescents (15–21 years) and in total available sequences. (PDF)

S1 Fig Antiretroviral susceptibility according to Stanford in 27 children (0–14) and 28 adolescents (15–21) samples with available sequence. (PDF)
