# Supplementary material for: High drug resistance levels could compromise the control of HIV infection in paediatric and adolescent population in Kinshasa, the Democratic Republic of Congo
Source: PLoS One. 2021 Apr 15;16(4):e0248835. doi: 10.1371/journal.pone.0248835 (PMC8049233; doi:10.1371/journal.pone.0248835)
Supplement: S1 Table — (PDF) [file pone.0248835.s001.pdf]

S1 Table. Patients carrying DRM to ARV families at study population: children (0-14 years), adolescents (15-21 years) and total.

	**Total seq.**	**Total DRM** n (%)	**Only Major-DRM** n (%)	**Major-DRM+Minor DRM to INI** n (%)	**Children seq.**	**Children** n (%)	**Adolescents Seq.**	**Adolescents** n (%)
**Single resistance**	55	7 (12.7)	7 (12.7)	7 (12.7)	27	2 (7.4)	28	5 (17.9)
**To NRTI**	49	0	0	0	23	0	26	0
**To NNRTI**	49	6 (12.2)	7 (14.3)	6 (12.2)	23	2 (8.7)	26	4 (15.4)
**To PI**	36	0	0	0	18	0	18	0
**To INI**	40	1 (2.5)	0	1 (2.5)	22	0	18	1 (5.6)
**Double resistance**	55	24 (43.6)	26 (47.3)	24 (43.6)	27	13 (46.4)	28	10 (35.7)
**NRTI+NNRTI**	49	22 (44.9)	26 (53.1)	22 (44.9)	23	12 (42.8)	26	10 (38.5)
**NRTI+INI**	34	1 (2.9)	0	1 (2.9)	18	0	16	1 (6.3)
**NNRTI+INI**	34	1 (2.9)	0	1 (2.9)	18	1 (5.6)	16	0
**Triple resistance**	55	6 (10.9)	3 (5.5)	5	27	3 (11.1)	28	3 (10.7)
**NRTI+NNRTI+PI**	36	4 (11.1)	3 (8.5)	3 (8.5)	18	2 (7.1)	18	2 (11.1)
**NRTI+NNRTI+INI**	34	2 (5.9)	0	2 (5.9)	18	1 (3.6)	16	1 (6.3)
**Quadruple resistance**	55	1 (1.8)	0	1 (1.8)	27	0	28	1 (3.6)
**PI+NRTI+NNRTI+INI**	26	1 (3.8)	0	1 (3.8)	15	0	11	1 (9.1)
**Summary resistance by ARV families**								
**No DRM**	55	17 (31)	18 (32.7)	17 (31)	27	9 (33.3)	28	8 (28.6)
**NRTI**	49	30 (61.2)	30 (61.2)	30 (61.2)	23	15 (65.2)	26	15 (57.7)
**NNRTI**	49	36 (73.5)	36 (73.5)	36 (73.5)	23	18 (78.3)	26	18 (69.2)
**PI**	36	5 (13.9)	3 (8.3)	3 (8.3)	18	2 (11.1)	18	3 (16.7)
**INI**	40	6 (15)	0	6 (15)	22	2 (9.1)	18	4 (22.2)

Legend S1 Table. n, number of patients; seq., sequences; PI, Protease Inhibitors; NRTI, nucleoside retrotranscriptase inhibitors; NNRTI, non-nucleoside retrotranscriptase inhibitors; INI, integrase inhibitors; DRM, drug resistance mutation; ARV, antiretroviral drugs.
