# Supplementary material for: High drug resistance levels could compromise the control of HIV infection in paediatric and adolescent population in Kinshasa, the Democratic Republic of Congo
Source: PLoS One. 2021 Apr 15;16(4):e0248835. doi: 10.1371/journal.pone.0248835 (PMC8049233; doi:10.1371/journal.pone.0248835)
Supplement: S2 Table — (PDF) [file pone.0248835.s002.pdf]

S2 Table. Drug resistance mutations in children (0-14 years), adolescents (15-21 years) and in total available sequences.

	Children Seq=27 (%)	Adolescents Seq=28 (%)	TOTAL Seq=55 (%)
DRM to NRTI	**15 (65.2) Seq=23**	**15 (57.7) Seq=26**	**30 (61.2) Seq=49**
**M184V**	11 (47.8)	11 (42.3)	22 (44.9)
**T215Y/F**	3/1 (17.4)	2/1 (11.5)	7 (14.3)
**K70R/N**	2/1 (13)	4 (15.4)	6/1 (14.3)
**M41L**	5 (21.7)	1 (3.8)	6 (12.2)
**L210W**	4 (17.4)	2 (7.7)	6 (12.2)
**E44D**	3 (13)	1 (3.8)	4 (8.2)
**D67N/E/G**	2/1/0 (13)	0/0/1 (3.8)	2/1/1 (8.2)
**K65R**	1 (4.3)	1 (3.8)	2 (4.1)
**L74I**	2 (8.7)	0	2 (4.1)
**K219N/E**	0/1 (4.3)	1/0 (3.8)	1/1 (4.1)
**T69D**	1 (4.3)	0	1 (2)
DRM to NNRTI	**18 (78.3) Seq=23**	**18 (69.2) Seq=26**	**36 (73.5) Seq=49**
**K103N/H/S**	9/1/0 (13)	10/0/1 (42.3)	19/1/1 (42.9)
**Y181C**	6 (26.1)	6 (23.1)	12 (24.5)
**G190A**	4 (17.4)	8 (30.8)	12 (24.5)
**V108I**	6 (26.1)	3 (11.5)	9 (18.4)
**K101E/H**	4/0 (17.4)	3/2 (19.2)	7/2 (18.4)
**H221Y**	3 (13)	3 (11.5)	6 (12.2)
**V106I**	3 (13)	3 (11.5)	6 (12.2)
**L100I/V**	1/0 (4.3)	2/1 (11.5)	3/1 (8.2)
**A98G**	2 (8.7)	1 (3.8)	3 (6.1)
**Y318F**	1 (4.3)	1 (3.8)	2 (4.1)
**K238T**	1 (4.3)	1 (3.8)	2 (4.1)
**Y188L, F227L, P225H**	0	1 (3.8)	1 (2)
DRM to PI	**2 (11.1) Seq=18**	**3 (16.7) Seq=18**	**5 (13.9) Seq=36**
Major	**1 (5.6)**	**2 (11.1)**	**3 (8.3)**
**M46I**	1 (5.6)	2 (11.1)	3 (8.3)
**I54V**	1 (5.6)	1 (5.6)	2 (5.6)
**I50V, I84V**	0	1 (5.6)	1 (2.8)
**I47V, V82M**	1 (5.6)	0	1 (2.8)
Minor	**2 (11.1)**	**1 (5.6)**	**3 (8.3)**
**K43T**	2 (11.1)	0	2 (5.6)
**L10F, I47L, Q58E, N83D**	0	1 (5.6)	1 (2.8)
Minor DRM to INI	**2 (9.1) Seq=22**	**4 (22.2) Seq=18**	**6 (15) Seq=40**
**E157Q**	0	2 (11.1)	2 (5)
**T97A**	1 (4.5)	1 (5.6)	2 (5)
**Q95K, G140GR**	1 (4.5)	1 (5.6)	1 (2.5)

Legend S2 Table. Seq., sequences; PI, Protease Inhibitors; NRTI, nucleoside retrotranscriptase inhibitors; NNRTI, non-nucleoside retrotranscriptase inhibitors; INI, integrase inhibitors; DRM, drug resistance mutation.
