# Supplementary material for: High drug resistance levels could compromise the control of HIV infection in paediatric and adolescent population in Kinshasa, the Democratic Republic of Congo
Source: PLoS One. 2021 Apr 15;16(4):e0248835. doi: 10.1371/journal.pone.0248835 (PMC8049233; doi:10.1371/journal.pone.0248835)
Supplement: S1 Fig — (PDF) [file pone.0248835.s003.pdf]

S1 Fig. Antiretroviral susceptibility according to Stanford in 27 children (0-14) and 28 adolescents (15-21) samples with available sequence

	ID	Protease Inhibitors								Nucleoside Reverse Transcriptase Inhibitors							Non-nucleoside Reverse Transcriptase Inhibitors					Integrase Inhibitors			
		ATV/r	DRV/r	FPV/r	IDV/r	LPV/r	NFV	SQV/r	TPV/r	ABC	AZT	D4T	DDI	FTC	3TC	TDF	DOR	EFV	ETR	NVP	RPV	BIC	DTG	EVG	RAL
CHILDREN (0-14)	16.RDC_P3										*				*	*		*		*					
	16.RDC_P5										*				*					*					
	16.RDC_P9										*				*					*					
	16.RDC_P10										*				*					*					
	16.RDC_P34										*				*	*		*		*					
	16.RDC_P35										*				*	*		*							
	16.RDC_P44										*				*					*					
	17.RDC_P45										*				*					*					
	17.RDC_P47										*				*			*		*					
	17.RDC_P48										*				*			*		*					
	17.RDC_P49										*				*					*					
	17.RDC_P50										*				*					*					
	17.RDC_P51										*				*	*		*		*					
	17.RDC_P52										*				*	*		*		*					
	17.RDC_P53										*				*			*		*					
	17.RDC_P58										*				*					*					
	18.RDC_P64										*				*					*					
	18.RDC_P74					*				*			*		*	*		*							
	18.RDC_P75									*	*				*			*		*					
	18.RDC_P76										*				*	*		*		*					
	18.RDC_P77					*				*					*										
	18.RDC_P84														*	*		*							

	18.RDC_P86					*				*					*	*									
	18.RDC_P87										*				*	*		*		*					
	18.RDC_P91										*				*	*		*		*					
	18.RDC_P93														*	*		*							
	18.RDC_P95									*					*	*		*		*					
ADOLESCENTS (15-21)	16.RDC_P4					*					*			*	*	*		*		*					
	16.RDC_P7										*				*	*		*		*					
	16.RDC_P13										*				*	*		*							
	16.RDC_P33										*			*	*	*		*		*					
	16.RDC_N37										*				*					*					
	16.CUN16														*	*		*							
	16.CUN22										*				*					*					
	17.RDC_P55										*				*					*					
	17.RDC_P56										*				*	*		*		*					
	17.RDC_P11										*				*					*					
	18.RDC_P65										*				*	*		*		*					
	18.RDC_P69										*				*	*		*		*					
	18.RDC_P71									*	*				*	*		*		*					
	18.RDC_P72										*				*	*		*		*					
	18.RDC_P73					*					*				*	*		*		*					
	18.RDC_P79										*				*	*		*		*					
	18.RDC_P80										*	*			*	*		*		*					
	18.RDC_P81					*					*	*		*	*	*		*		*					
	18.RDC_P83										*				*	*		*		*					
	18.RDC_P85										*	*			*	*		*		*					
	18.RDC_P89									*	*				*	*		*		*			*		
	18.RDC_P90										*				*	*		*		*					

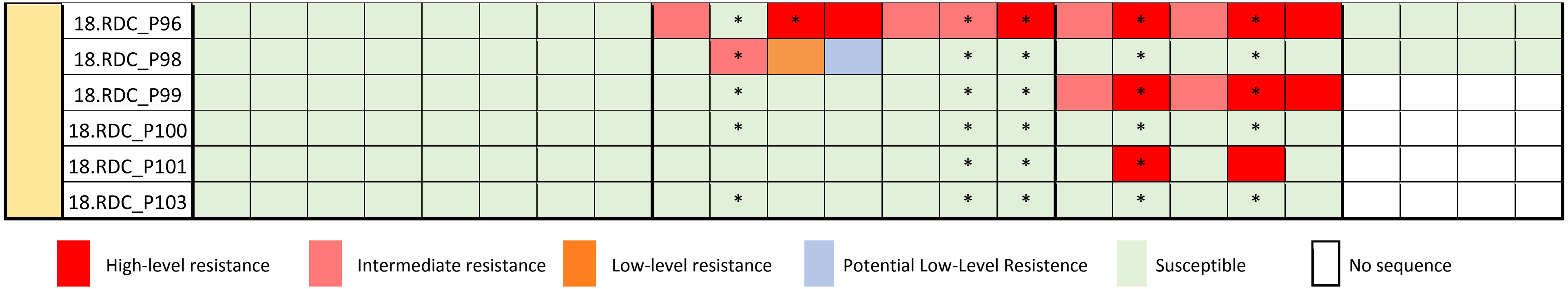

Legend S1 Fig. *ARV experience. Patients´ ID codes were provided in laboratory after sample reception to maintain their anonymity. ATV/r, Atazanavir/ritonavir; DRV/r, Darunavir/ritonavir; FPV/r, Fosamprenavir/ritonavir; IDV/r, Indinavir/ritonavir; LVP/r, Lopinavir/Ritonavir; NFV, Nelfinavir; SQW/r, Saquinavir/ritonavir; TPV/r Tipranavir/ritonavir; ABC, Abacavir; AZT, Zidobudine; D4T, Estavudine; DDI, Didanosine; FTC, Emtricitabine; 3TC, Lamivudine; TDF, Tenofovir; DOR, Doravirine; EFV, Enfavirez; ETR, Etravirine; NVP, Nevirapine; RPV, Rilpivirine; BIC, Bictegravir; DTG, Dolutegravir; EVG, Elvitegravir; RAL, Raltegravir.
